# Case Department

**Published:** 1888-04

**Authors:** 


### CASE DEPARTMENT.

I. Emphysema of Neck Due to Sloughing of Lung Tissue.—The subject of the above condition was a large black draft mare nine years old, which had been suffering from an attack of influenza complicated with inflammation of the lower part of the right lung. On the tenth day of attack the breath became fœtid and coughing violent and paroxysmal, large quantities of brownish colored fluid and semi-fluid matter being discharged both through the mouth and nostrils.

The cough continued very violent for some time, and on the fourteenth day an emphysematous swelling appeared on the neck, being most marked round the region of the parotid glands, where it was so prominent that the angles of the jaw and base of the ears were quite hidden.

The swelling did not extend far backwards, but could be traced right down the course of the trachea into the chest. Over the upper part of the right lung, where hitherto the respiratory murmurs had been abnormally loud, auscultation revealed diminished lung sound, so I judged this part of the lung was like the neck, in an emphysematous state.

A little below the level of the larger bronchial tubes was a spot about the size of the hand, where a faint sighing or whistling sound could be heard, indicating the seat of sloughing, and where also the gas had gained entrance to the interstitial tissue, being forced in by the violence of coughing, afterwards infiltrating its way through the cellular tissues surrounding the trachea, and accumulating in the comparatively loose structures around the parotid glands.

I adopted no special treatment, and in five days the enlargement had disappeared.

During the height of the disease, this mare had a temperature of Fah. 105.5°, pulse 88 and respirations 62.

In addition to the usual attention to pure air, warm clothing and suitable food, the treatment of this case consisted in bi-daily doses of nitrate and chlorate of potash, combined with a half a dram of quinine and three ounces of good whiskey, also frequent inhalations of steam medicated with oil of turpentine. The animal made a good recovery.

This mare was one of two horses working together, the horse with which she worked being laid off with the same disease (uncomplicated) two days previously.

The infection was introduced into the stable twenty-five days before with a Canadian mare which was unwell at the time she was bought, and within five days developed the disease.

This, then, would give an incubative period of about twenty-four days.

GEORGE N. KINNELL, M. R. C. V. S.
Pittsfield, Mass.

II. THE USE OF CHLORAL HYDRAS AS AN ANTISPASMODIC.—I have been very successful in the use of chloral in my practice. In cases of inverted uterus in cows, after replacement, a dose of oz. i to oz. ij being sufficient to relieve the difficulty. In mania purpueralis and in colic the same drug was used with success. I have also used it on the horse with equally good results in cases of acute mania, spasmodic colic, azaturia, tetanus and trembling produced by fright, combining it with linseed oil and aloes, 1 pint of the former to oz. ij of the latter in cases of colic. In tetanus, in addition to the chloral, used as an enema with an infusion of tobacco, bromide of potass. and belladona every four hours. I used hypodermic injections of nitrate of amyl, a drachm night and morning. Five cases in succession recovered under this treatment.

INVERSION OF UTERUS WITH PARTURIENT FEVER.—I was called in the case of a cow which had just given birth to a calf. Found her suffering with parturient fever and prescribed Prof. D. McIntosh's remedy :

℞ Mag. sulph. lb i.
Zingiber pulv. oz. ij.
Gamboge dr. ij.
M. ft. chart. No. 1. Sig. one dose.

Also ℞ spts. ammon Ar. oz. x.
Spts. nitri dulc. oz. xx.

M. sig. oz. iij every half hour for two and one-half hours, then oz. iij every hour.

In addition mustard friction to the loins. After third day hypodermic injections of ½ grain of strychnia in the neck, night and morning.

DEATH OF A COW CAUSED BY A PIECE OF WIRE PENETRATING THE HEART.—The animal was first taken with diarrhœa, a swelling on brisket, capricious appetite. It was an impossibility to control the diarrhœa. I gave everything recommended in the U. S. Dispensatory without success, finally she dropped dead, and I performed autopsy, and found a piece of wire shaped thus L, 8 inches long, which had penetrated the heart. P. Z. COLSSON, V.S., ONT.
Mobile, Ala.

III. ŒSOPHAGUS OF A HORSE CARRIED AWAY ENTIRE BY SLOUGHING.—On June 5 I was called to see a mare that had been drinking lye. I prescribed the usual remedies. For ten days she appeared well and grazed. On June 15 I was called again, found her with no desire to eat or drink. On attempting to drink the water, instead of it passing into the stomach, it seemed to fill up the æsophagus, as the parts bulged out plainly on the left side of the neck and caused choking, coughing and efforts of vomiting, grass and water coming up through the nostrils. The enlargement in the æsophagus remained, but I was able to reduce it somewhat by manipulation, and she seemed to be entirely relieved. After this she took food and drink again with the same results, but this time I was unable to relieve her by manipulation. I diagnosed a stricture of the æsophagus caused by ulceration and cicatrization resulting from the action of the lye. I caused the animal to be brought to my infirmary, and as I could find no case on record to guide me, I resolved on the following plan of treatment: I united two horse catheters so as to form a continuous tube and passed it back through the nostril into the æsophagus. I then attached my pump and injected water into the mass, which, after some coughing and choking and efforts at vomiting, came away. I was then enabled to pass the instrument into the stomach, being obliged, however, to use a wire stillet. I then attempted to feed her on sloppy food, but after five days, to prevent starvation, I injected through the probang devised as above, milk and eggs well beaten up together with water; this I continued twice daily for seven days.

The fæcal matter becoming offensive I removed her to an outhouse, keeping her there until she was able to eat grass without difficulty. Twelve days after her reception at the infirmary my attention was attracted by an object in the fæces, which on examination proved to be the mucous membrane of the æsophagus of cylindrical form, intact with the exception of a hole six inches from one end. It measured twenty-six inches in length. On the 11th of July the animal was discharged cured. During the summer she suffered from a few paroxysms of choking, but was able to relieve herself. This specimen has been on exhibition at the Ontario Veterinary College this winter. H. E. Rowell, V.S.,
Albion, N. Y.

IV. Bovine Monstrosity.—In a cow eight years old, ten days past term, found fœtal envelopes presented. I labored seven hours in effecting delivery, and accomplished the same by skinning the right fore leg and removing; also disarticulated right hind leg at coxo-femoral joint. Monstrosity belonged to the order named by Gurlt as schistocormus degree schistepigastrico-sternalis, meaning non-closure of thorax and abdomen. The diaphragm was absent, and ribs were turned back over vertebra instead of enclosing thorax. Fœtal heart-beat could be readily felt. Could have delivered without embryotomy if it had not been for leg deformity of fœtus.

Oblique Fracture of Radius in Five-Year Old Gelding.—Fracture caused by a partition log falling on limb while horse was lying down. I adjusted the fracture, which was close to carpus, twenty-four hours after the accident by using tin and wood splints and plaster paris bandage. Placed horse in slings. Bones united. He had been in sling sixty-one days when I discovered symptoms of tetanus. Removed splints to investigate, and found that a tin splint had chafed carpus near inner border of trapesium. Treatment consisted thenceforth of bellad. ext. on sore. Tetanic symptoms developed rapidly. Hydrocyanic acid, cal. hyd. and potassa brom. given; absolute quietness ordered; symptoms abated; horse improving rapidly, but in a paroxysm he caused a severe inguinal hernia by bearing his weight in the sling. I could not dispense with the sling on account of the fractured leg. I destroyed him when hernia occurred. Necropsy showed perfect union of fracture. The sling was the indirect cause of his destruction, as he would ultimately have recovered had the hernia not occurred.

Bovine Metrotomy.—Cow five years old, poorly nourished, had given birth to her third calf. The uterus became inverted a few hours after delivery owing to severe after pains. The cow was lying down and unable to rise, and the efforts of the neighbors to replace the uterus were unsuccessful. The uterus was frightfully lacerated. Advised owner of condition, and obtained permission to amputate, which I considered to be the only way to save the cow. The operation was performed at once by applying a double ligature as far up as the base of the inverted mass would allow, without including meatus, and one-half of the base being included in each ligature, the attraction being made by four strong men, and heavy waxed silk used. The mass was then removed by the knife. Hypodermic injections of morphia were given and repeated as symptoms indicated very severe pain. The cow regained standing position, kicked, stamped, jumped and moaned for twelve hours after amputation. Iodoform and carbolized oil were used for dressing, and the sustaining treatment adopted internally. On the sixth day the slough of the ligated portion took place. The cow has done well since, having given milk constantly for two years. As both cornuæ were inverted and both ovaries were removed, there has been no œstrum.

Exostosis in Cervical Vertebræ of Horse.—On April 5, 1886, I was called to see a trotting gelding and found the following condition: Well nourished, pulse 48, temp. 100° F., appetite good, bowels regular and normal appearance of visible mucous membranes. History. Had been previously a trotter in several races. During the period of training the actions of the horse were noticed as rather strange when walking or jogging. He was used at this time as a road horse and continued to stagger or totter at times. Recently after returning from a long ride on a warm day and drinking freely of cold water he was seized with well-marked rigors and showed symptoms of laminitis, and was treated for the same by a local practitioner. Shortly afterward a swelling made its appearance on each side of the neck, which gradually subsided.

Then the animal began to show signs of weakness in and loss of control of the anterior extremities, and notwithstanding treatment grew worse. Paralysis of the posterior extremities, stumbling and falling as in the loss of co-ordination, observed in Locomotor Ataxia.

I diagnosed a spinal lesion at cervical region and gave fatal prognosis.

The horse was destroyed, and on examination I found an osseous deposit between the fifth and sixth cervical vertebræ, extending between the articular processes of the left side in such a manner as to cause pressure on the spinal cord. On section of the exostosis I found an abscess in the centre containing about ½ oz. of bloody pus. This lesion may have been caused by an external injury, or more probably was due to the fact of being checked very high during his trotting career—as is customary—thus exciting inflammatory action with the above consequences.

ABDOMINAL PUNCTURE.—Four-year-old colt ran away and jumped on a sharp iron post, causing an incised wound of five inches at umbilicus. The colt was led five miles to infirmary, the weather being very cold. On examination I found a portion of the colon protruding and purple from exposure to cold. It was evidently a hopeless case, but at the earnest solicitation of the owner the colt was cast, and hernia being strangulated, opening was enlarged, bowel bathed with tepid water, carbolized and returned, abdominal opening closed with twenty carbolized gut sutures, and cutaneous opening with large clamp. oz. i powd. opii. given every three to four hours, till action of bowels stopped. I had the satisfaction of discharging this colt cured.

CHARLES C. MCLEAN, *Veterinarian,*
Meadville, Pa.

V.—TORSION OF THE NECK OF THE UTERUS.—I was called to attend a cow which the owner stated had been in labor during the night previous, but as yet there was no presentation of the calf. On my arrival I found the cow lying down almost exhausted, the labor efforts being very feeble On introducing my hand into the vagina for the purpose of examining the condition, I discovered the uterus to be twisted, so that I was unable to enter the neck further than a finger's length. I could feel the calf's nose and fore legs in the twisted neck. I had a number of large bundles of straw placed under the hind parts so as to raise them quite high, that the calf might slip forward, and enable me the better to examine the os and neck as well as to discover the direction of the twist. I had the animal turned from right to left, at the same time keeping my hand in the neck of the uterus ; this indicated to me the direction of the twist, as I could feel the twisted neck tighten upon my hand, showing that the uterus had been turned in the same direction. I therefore had the animal put back into the former position and drawn to the left side of the stall, tying her fore legs together, keeping her on her left side. I had one man take charge of the head, so that the horns might not be injured, while three others took charge of the body. I had the cow turned completely over from left to right until she rested upon the left side again, making a complete revolution. After this I was enabled to deliver the calf alive without any trouble. With a little stimulating tonic and warm clothing to the cow, in two days she was in her normal condition.

JOHN LINDSAY, D. V. S.,
Huntington, Long Island.

VI.—ABSCESS IN THE BRAIN OF A HORSE FROM STRANGLES. (Thierry—*Recueil de Med. Vet.*)—The following case is interesting from its rarity and from the fact that the diagnosis and prognosis were accurately made before the death of the animal. A young horse affected with strangles had had multiple abscesses of the submaxillary space, the parotid region and the cheek ; eight days after M. Thierry had pronounced the animal recovered, he developed a new trouble ; marked drowsiness, slow mastication and deglutition, dulled vision, feebleness of the legs, turning to the left. Diagnosis—abscess of the right cerebral lobe. Autopsy—In the cortical portion of the right cerebral lobe, a large abscess with thick resisting walls completely independant of the lateral ventricle.—Ed. Notes.

Any grave organic disturbance occuring within the two weeks following the apparent recovery of any case of strangles, however mild and free from complications it may have been, should lead promptly to suspicion of the formation of abscess. The following case is from the notes of Mr. Ridge, a veterinary student at the University of Pennsylvania.

A ten year old sorrel, 16½ hands, of great value, contracted the strangles in May, 1887, from a pair of recently purchased horses. Made apparently a perfect recovery. In June had an attack of light colic, which was followed by a progressive anæmia. Diagnosis was made by Mr. Ridge, of mesenteric abscess, an unfavorable prognosis given and in August the diagnosis and prognosis were confirmed by the autopsy. R. S. H.

